# Temporal Summation of Pain Unpleasantness Is Increased in Borderline Personality Disorder

**DOI:** 10.1002/ejp.70042

**Published:** 2025-05-22

**Authors:** Annette Löffler, Dieter Kleinböhl, Sylvia Steinmann, Sabine C. Herpertz, Ute Habel, Robin Bekrater‐Bodmann, Herta Flor

**Affiliations:** ^1^ Institute of Cognitive and Clinical Neuroscience, Central Institute of Mental Health, Medical Faculty Mannheim Heidelberg University Mannheim Germany; ^2^ Department of Psychosomatic Medicine and Psychotherapy, Central Institute of Mental Health, Medical Faculty Mannheim Heidelberg University Mannheim Germany; ^3^ Department of Psychiatry, Psychotherapy and Psychosomatics Uniklinik RWTH Aachen Aachen Germany; ^4^ Scientific Center for Neuropathic Pain Aachen SCN^AACHEN^ Uniklinik RWTH Aachen Aachen Germany; ^5^ Department of General Psychiatry, Medical Faculty, Center for Psychosocial Medicine Heidelberg University Heidelberg Germany

**Keywords:** affective pain perception, borderline personality disorder, pain paradox, RIII‐reflex, temporal summation of pain

## Abstract

**Background:**

Borderline personality disorder (BPD) is characterised by deficient regulation of emotions and is associated with reduced pain sensitivity, which has been related to self‐injury and dissociation. BPD can therefore be used as a model to better understand pain‐modulating mechanisms and their association with affective processing. However, studies assessing pain‐modulating processes in BPD are sparse.

**Methods:**

This study investigated temporal summation (TS) of pain intensity and unpleasantness, as well as TS of the RIII‐reflex as a marker for spinal nociceptive processing in 24 participants with BPD compared to 24 non‐clinical controls (NCC).

**Results:**

Our main result showed that TS of pain unpleasantness, but not TS of pain intensity, was significantly increased in BPD compared to NCC, whereas we replicated higher pain thresholds in BPD compared to NCC. There was no significant correlation between pain threshold and TS of pain intensity or TS of pain unpleasantness in BPD. Moreover, correlative findings suggest a mutual dependence of spinal processing, temporal summation of pain and stimulus intensity in NCC, but not in participants with BPD.

**Conclusions:**

The combination of reduced pain sensitivity in terms of heightened pain threshold and enhanced TS of pain unpleasantness might explain the so‐called pain paradox, describing that individuals with BPD are both hyposensitive to acute pain and more prone to develop chronic pain. Different mechanisms might underlie heightened pain thresholds and increased TS of pain unpleasantness based on a complex interaction of altered ascending and descending mechanisms.

**Significance Statement:**

The results of this study provide evidence that temporal summation of pain unpleasantness is increased in individuals with borderline personality disorder compared to non‐clinical controls. These data suggest that altered pain perception in BPD is composed of several processes, extending beyond well‐known pain insensitivity.

## 1 Introduction

Borderline personality disorder (BPD) is a frequent mental disorder with a lifetime prevalence of about 6% (Grant et al. 2008). Reduced pain sensitivity is a feature of BPD that has been related to non‐suicidal self‐injurious behaviour (NSSI) (Koenig et al. 2016), which is common in BPD (Zanarini et al. 2008). Reduced sensitivity to experimental pain in BPD was shown in various studies for several pain modalities (e.g., Bekrater‐Bodmann et al. 2015; Ludäscher et al. 2007; Magerl et al. 2012). A recent meta‐analysis confirmed reduced pain sensitivity in BPD compared to non‐clinical controls (NCC) (Fales et al. 2021). BPD can therefore serve as a model to study the mechanisms underlying hypoalgesia (Magerl et al. 2012). However, the mechanisms underlying reduced pain sensitivity in BPD are still largely unknown (Bekrater‐Bodmann 2021).

A pain‐modulating mechanism that might be involved in altered pain perception in BPD is temporal summation (TS) of pain. TS of pain refers to an increase in pain when noxious stimuli of constant physical intensity are repeatedly delivered with frequencies above 0.3 Hz and is considered a perceptual correlate of wind‐up, a spinal excitatory nociceptive process (Kleinböhl et al. 2006; Price 1972). TS of pain depends on *N*‐methyl‐D‐aspartate (NMDA) receptor activation, with NMDA antagonists such as ketamine reducing TS of pain (Eide 2000). Interestingly, NMDA antagonists also evoke dissociation (e.g., Krystal et al. 1994), a diagnostic feature of BPD (American Psychiatric Association 2013), characterised by reduced pain sensitivity (e.g., Ludäscher et al. 2007). NMDA receptor dysfunction might therefore play a role in altered pain perception in BPD (Bekrater‐Bodmann et al. 2015; Grosjean and Tsai 2007), involving pain insensitivity and reduced TS of pain. In fact, heat pain threshold and TS of pain intensity were found to be negatively correlated in BPD, but not in NCC, suggesting that reduced TS of pain might contribute to hypoalgesia in BPD (Defrin et al. 2020). Previous studies failed to provide evidence for reduced TS of pain in BPD compared to NCC for thermal (Defrin et al. 2020) or mechanical stimuli (Ginzburg et al. 2018). However, these studies focused on TS of pain intensity and did not evaluate TS of pain unpleasantness, which is important for the understanding of altered pain perception as both pain components, that is, the sensory and affective component, are processed in different brain areas. Particularly in the context of BPD, it is important to assess both pain components, since previous results of imaging studies suggest altered prefrontal–limbic coupling in BPD that underlies abnormal processing of especially the affective pain component (Schmahl and Baumgärtner 2015). In addition to the perceptual level of pain, the present study further aimed to investigate the spinal nociceptive processes underlying TS of pain, which have received little attention in previous research on pain modulating processes in BPD.

In the present study, we assessed electrical pain threshold and TS of pain intensity and unpleasantness in participants with BPD and NCC. We further measured the RIII‐reflex, a widely used neurophysiological measure of spinal nociceptive processing in humans (Sandrini et al. 2005). We expected reduced TS of pain, especially of pain unpleasantness, and reduced TS of the RIII‐reflex in participants with BPD compared to NCC. For BPD, we expected a negative association between TS of pain and clinical markers, in terms of dissociation and NSSI.

## 2 Methods

### 2.1 Sample

We included data of *n* = 24 female participants with a current diagnosis of BPD and *n* = 24 female NCC. Results of an independent samples *t*‐test revealed no significant age differences between both groups (BPD: *M* = 29.25 years, SD = 7.70; NCC: *M* = 30.42 years, SD = 8.46), *t*_46_ = 0.50, *p* = 0.62.

Participants were recruited through a central recruitment unit of a Clinical Research Unit on BPD (Schmahl et al. 2014). Sample size selection was based on previous studies on pain perception or modulation in BPD (e.g., Chung et al. 2020; Defrin et al. 2020; with sample sizes of 25 or 22 in the clinical groups, respectively). Clinical diagnosis according to the Diagnostic and Statistical Manual for Mental Disorders IV (DSM‐IV) (American Psychiatric Association 2000) was made by trained mental health personnel using the Structured Clinical Interview for DSM‐IV Axis I Disorders (SCID‐I, Wittchen et al. 1997) to assess comorbid Axis I Disorders. SCID‐I data of one participant is missing. The International Personality Disorder Examination (IPDE; Loranger et al. 1997) was used for BPD diagnostics. Participants with BPD had to meet five or more of the BPD IPDE criteria within the last 2 years prior to study participation, and at least one of these criteria had to begin during childhood or adolescence. All participants were fluent in German and all but three participants were right‐handed (two ambidextrous in the BPD group, one in the NCC group) as assessed with the Edinburgh Handedness Inventory (Oldfield 1971). Participants with BPD discontinued their regular medication for at least 2 weeks prior to study participation with the exception of selective serotonin reuptake inhibitors, SSRI, for which discontinuation is not recommended given the evidence for adverse physical and psychological symptoms that may occur with its discontinuation (Fava et al. 2015), and for 2 days prior to participation for on‐demand medication (such as sedative‐hypnotics or benzodiazepines). Three participants with BPD reported taking SSRI during study participation; data on one participant are missing. The study was approved by the ethics committee of the Medical Faculty Mannheim of the University of Heidelberg (2014‐609N‐MA) and complied with the Declaration of Helsinki. All participants provided written informed consent and were compensated for participation with 26€.

A priori, we excluded participants with scars in the area of the ankle or back of the thigh of the right leg (regardless of whether the scar is the result of self‐injurious behaviour or other reasons) to avoid reduced sensitivity in the stimulated body part or problems with electromyographic (EMG) recordings. Further exclusion criteria were life‐time diagnosis of bipolar I disorder or schizophrenia, insufficient language comprehension, body mass index < 16.5, substance abuse within the last 2 months, fibromyalgia, serious physical illness, severe brain diseases or concussion and pregnancy. Prevalence of comorbid life‐time and current mental disorders as well as a clinical characterisation of the BPD sample are given in Table 1. A history of mental disorders was an exclusion criterion for the NCC group.

**TABLE 1:** Prevalence of comorbid mental disorders in participants with borderline personality disorder and clinical characteristics of the participants.

Prevalence of comorbid mental disorders in BPD (*n* = 23)	Current *n* (%)	Lifetime^a^ *n* (%)
Major depressive disorder	7 (30)	18 (78)
Post‐traumatic stress disorder	4 (17)	6 (26)
Eating disorders	2 (9)	13 (57)
Other mental disorders (only current)	10 (43)	—
More than one mental disorder (only current)	9 (39)	—

Clinical characteristics	NCC (*n* = 24)	BPD (*n* = 24)	Test statistics
	*M* ± SD Mdn; IQR	*M* ± SD Mdn; IQR	
Symptom severity (BSL‐23)	0.09 ***±*** 0.09 0.07; 0.17	1.34 ***±*** 0.84 1.24; 1.51^c^	*z* = −5.74, *p* < 0.001, *r* = 0.85
Frequency NSSI last month^b^	—	6.64 ***±*** 5.88 4.00; 7.00	—
Trait dissociation (FDS)	2.96 ***±*** 2.20 2.27; 2.67	20.57 ***±*** 12.56 20.45; 16.14^d^	*z* = −5.36, *p* < 0.001, *r* = 0.78
Depressiveness (BDI)	1.96 ***±*** 2.51 1.00; 3.25	18.86 ***±*** 10.65 22.00; 13.00	*z* = −5.15, *p* < 0.001, *r* = 0.76
Trait anxiety (STAI)	31.88 ***±*** 6.26 29.50; 9.50	65.00 ***±*** 7.43 67.00; 9.50^d^	*t*_45_ = −16.52, *p* < 0.001, *d* = 4.82
State anxiety (STAI)	29.33 ***±*** 4.05 29.50; 5.00	52.86 ***±*** 12.70 53.00; 20.50^d^	*t*_44_ = −8.62, *p* < 0.001, *d* = 2.50

*Note:* Data of the Structured Clinical Interview for DSM‐IV Axis I Disorders (Wittchen et al. 1997) were missing for one participant with BPD.

Abbreviations: BDI, Beck Depression Inventory (Hautzinger et al. 1995); BPD, participants with borderline personality disorder; BSL‐23, Borderline Symptom List (Bohus et al. 2009); FDS, Fragebogen zu Dissoziativen Symptomen [Questionnaire of dissociative symptoms] (Freyberger et al. 1999) German version of the Dissociative Experience Scale (Bernstein and Putnam 1986); IQR, interquartile range; *M*, mean; Mdn, median; *n*, number; NCC, non‐clinical controls; NSSI, non‐suicidal self‐injury; SD, standard deviation; STAI, State–Trait‐Anxiety Inventory (Laux et al. 1981).

^a^ Including current comorbidities.

^b^ Reported only from those participants who performed NSSI within the last year prior to study participation at all (*n* = 21). None of the NCCs reported NSSI.

^c^ *n* = 22.

^d^ *n* = 23.

Another *n* = 9 female participants—five with BPD and four NCC—were recruited but either terminated the experiment prematurely (*n* = 5 with BPD and *n* = 2 NCC, due to intolerance of electrical stimulation or severe dissociation during the experiment) or reported former injury in the stimulation area *and* were statistical outliers for pain threshold (at least 2 SD higher than the group mean), indicating abnormal nociceptive processing (*n* = 2 NCC); these participants were excluded from the analyses.

From the final sample (*N* = 48), *n* = 8 participants (five with BPD and three NCC) reported former pain episodes or injuries (e.g., torn ligament or ankle sprain) in the stimulation area but none of these participants was a statistical outlier for pain threshold or TS of pain. Another *n* = 2 participants (one with BPD and one NCC) reported regular pain (i.e., several days a month) in terms of back pain or migraine.

### 2.2 Psychological Assessment

We used the short version of the German Borderline Symptom List (BSL‐23; Bohus et al. 2009) to assess general symptom severity. The questionnaire asks the participants to evaluate their symptoms (e.g., ‘I felt helpless’) during the past week on a 5‐point Likert scale ranging from 0 (‘not at all’) to 4 (‘very strong’). The mean score of all items serves as indicator for symptom severity with higher values indicating higher symptom severity. The questionnaire is widely used and has been shown to have good psychometric properties (Bohus et al. 2009). For the assessment of depressiveness, we used the German version of the Beck Depression Inventory (BDI; Hautzinger et al. 1995). In this questionnaire, participants rate 21 different symptoms (e.g., sadness) regarding how closely it corresponds to how they have felt in the last week. The overall sum score serves as indicator for severity of depressiveness with values ranging from 0 to 63 and higher scores indicating higher depressiveness. The questionnaire can be used in clinical conditions and has strong psychometric support (Hautzinger et al. 1995). The German version of the State–Trait‐Anxiety Inventory (STAI; Laux et al. 1981) was used to assess level of anxiety. The 40 items of the questionnaire consist of 20 items asking participants to rate their current emotional state (e.g., ‘I am nervous’) to evaluate state anxiety (i.e., anxiety as a temporary emotional state which varies in intensity over time and situations) on a 4‐point Likert scale, ranging from ‘not at all’ to ‘very much’. The other 20 items (e.g., ‘I am a steady person’) assess trait anxiety (i.e., anxiety as personality trait) on a 4‐point Likert‐scale, ranging from ‘almost never’ to ‘almost ever’. For both the state and trait subscale, the sum scores range from 20 to 80 with higher values indicating higher anxiety. The STAI is considered a standard instrument to assess anxiety and has shown high psychometric quality (Laux et al. 1981). For the assessment of trait dissociation we used the Fragebogen zu Dissoziativen Symptomen (FDS; Freyberger et al. 1999), which is the German adaptation of the Dissociative Experience Scale (Bernstein and Putnam 1986). The questionnaire assesses dissociative symptoms, including depersonalization, derealization, amnesia and conversion. In addition to these subscale, a total score from all 44 items, ranging from 0 to 100, serves as indicator for general trait dissociation with higher values indicating higher trait dissociation. Frequency of NSSI was assessed only for those participants who had reported NSSI at all with in the last year prior to study participation (*n* = 21). We assessed self‐reported number of self‐injurious acts within the last month prior to study participation. Participants reported number of acts for 13 specific kinds of self‐injurious behaviour (e.g., cutting, burning, pulling out hair and so forth) as well as for ‘other behaviours’ to capture any behaviours that did not fit into the predefined categories. The sum score of all categories was used as indicator for frequency of NSSI. These data were assessed on a separate day and were missing for *n* = 1 participant with BPD for FDS, NSSI and trait anxiety, as well as for *n* = 2 participants with BPD for BSL and state anxiety.

**FIGURE 1:**
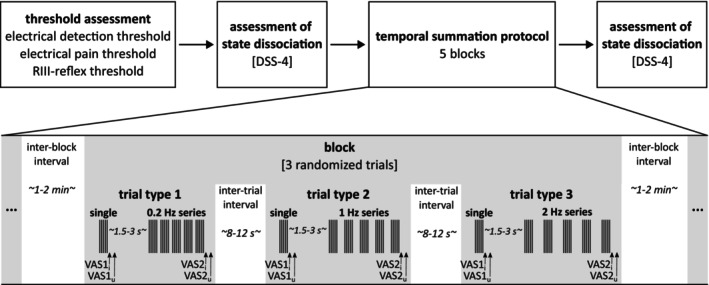
Experimental design. At the beginning of the session electrical detection threshold, electrical pain threshold and the RIII‐reflex threshold were measured. A temporal summation protocol was used to generate and measure temporal summation of pain during repetitive electrical stimulation. The stimulus was a pulse train, consisting of a series of five single pulses of 1 ms duration, with a base rate of 250 Hz. One trial consisted of a single pulse train and a series of five pulse trains delivered with 0.2, 1, or 2 Hz. Two visual analogue scales were presented immediately after the single pulse train (VAS1) and the 5th pulse train of a series (VAS2) to assess perceived pain intensity (VAS1_i_ and VAS2_i_) and unpleasantness (VAS1_u_ and VAS2_u_) of the respective stimulus. Time between the rating of the single pulse train and onset of the subsequent series was randomised between 1.5 and 3 s. The experiment consisted of five blocks with three randomised trials each. The inter‐trial interval was randomised between 8 and 12 s, the inter‐block interval was randomised between 1 and 2 min. Stimulus intensities were preset to 150% of electrical pain threshold (EPT). Before and after the temporal summation protocol state dissociation was assessed by using the German short version of the Dissociation‐Tension Scale acute (DSS‐4; Stiglmayr et al. 2009).

During the experiment, we assessed state dissociation using the German short version of the Dissociation‐Tension Scale acute (DSS‐4; Stiglmayr et al. 2009) immediately before the temporal summation protocol (see Figure 1). The DSS‐4 consists of four items assessing somatoform dissociation, analgesia, depersonalization and derealization and can be used repeatedly during experimental settings. The mean score ranges from 0 to 9, with higher values indicating higher dissociation. Additionally, we assessed the DSS‐4 immediately after the temporal summation protocol to explore the association between TS of pain and change in dissociation from pre to post painful stimulation. This might be interesting in the context of NSSI, as it has been shown that most individuals with BPD perform NSSI (i.e., self‐infliction of pain) to reduce aversive inner tension associated with dissociation (Kleindienst et al. 2008). Data were missing for *n* = 1 participant with BPD after painful stimulation.

### 2.3 Electric Stimulation and EMG Recording

Before attaching the electrodes, electrode sites were cleaned with surgical spirit and abraded with V17 Abralyt 2000 (Easycap GmbH, Herrsching, Germany) to achieve impedances of less than 10 kΩ. The external retro‐malleolar pathway of the sural nerve of the right leg was stimulated percutaneously using a Nicolet surface bar electrode (bipolar stimulating electrode of 8 mm diameter with 30 mm interelectrode distance) that was applied with anode inferior (e.g., Rhudy and France 2007). To ensure that the sural nerve was stimulated, a position on the ankle was chosen where electrical stimulation was felt on the outer edge of the foot by the participant. After attaching the electrode, the ankle was fixed at 90° (Sandrini et al. 2005) using a SAM splint (SAM Medical, Tualtin, Oregon, USA) and a bandage. Electrical stimuli were generated by an electrical stimulator (Digitimer, DS7A; Digitimer Ltd., Welwyn Garden City, UK) controlled by Presentation (v17.0; Neurobehavioral Systems Inc., Albany, CA, USA) and consisted of standard pulse trains of five rectangular pulses (each of 1 ms duration) delivered at 250 Hz (Terry et al. 2011). These pulse trains are typical for RIII studies and have been shown to be most efficacious to evoke an EMG response (Sandrini et al. 2005). Since it is extremely brief, one pulse train is perceived like a single stimulus by the participant. In order to record biceps femoris activity of the right leg, two surface electrodes (Neonatal ECG electrode; Philips HP Agilent, Palo Alto, California, USA) were attached over the muscle belly of the brevis head. Further, a ground electrode was attached above the tibia, midway between the knee and ankle. To achieve muscle relaxation during the experiment, participants were seated comfortably on an examination table with the knee supported by a knee roll (knee flexed at 120°–130°), the ankle fixed at 90° (see above) and the upper body reclined (angle of approx. 100° between the upper body and the upper leg) (Sandrini et al. 2005). The legs were covered with a blanket to prevent them from cooling down and to make lying on the bed with bare legs less uncomfortable. EMG activity was amplified using a bioamplifier V75‐04 of a LabLinc V System (Coulbourn Instruments, Allentown, PA, USA) with a signal bandwidth of DC—1 kHz. The signal was processed using a CED 1401 Power analog‐to‐digital converter and Spike2 version 2.13 software with a sampling rate of 5 kHz (both: Cambridge Electronic Design Lfg, Cambridge, England). In one participant with BPD, the left instead of the right leg was stimulated because the participant reported reduced sensibility in the innervation area of the sural nerve after a herniated disk in the left but not the right leg.

### 2.4 Threshold Assessment

Before the main experiment started, electrical detection threshold (EDT), electrical pain threshold (EPT) and RIII‐reflex threshold (RT) were assessed in separate runs by stimulating the external retro‐malleolar pathway of the sural nerve of the right leg using single pulse trains and three ascending–descending staircases of electric stimuli. The interval between two pulse trains varied randomly between 8 and 12 s to reduce predictability and habituation (Terry et al. 2011). In all participants, we started with the assessment of EDT, followed by EPT and RT assessment. For the RT procedure, electric stimulation started with 0 mA. The EMG signal was analysed online and the stimulus intensity of the following pulse trains was increased when there was no valid reflex present and decreased when there was a valid reflex present in response to the preceding stimulus. However, post hoc offline analysis revealed that due to slow drifts in the EMG signal, the results of the online analysis might have been misleading, and thus RT is not reported. Since stimulation intensity was adjusted depending on the result of EPT and a DC correction was applied to the data for all offline analyses of the EMG data (see below), the described issue did not influence the reported results.

Participants were instructed to verbally indicate when they perceived the electrical stimulation (EDT) and as soon as the stimulus was perceived as just painful (EPT). For the assessment of EDT, electric stimulation started with 0 mA. Stimulation intensities of the following pulse trains were then manually increased in 2 mA steps until perception was reported. The current was then manually decreased in 1 mA steps until it was no longer perceived by the participants. The next two ascending‐descending staircases continued with 1 mA steps. EDT was defined as the average stimulation intensity (mA) of the 2 peaks and 2 troughs of the last two ascending‐descending staircases. For EPT assessment, starting from calculated EDT, electrical stimulation intensities of the following pulse trains were manually increased in 2 mA steps until it was perceived as just painful. The current was then manually decreased in 1 mA steps until it was no longer perceived painful, followed by two ascending‐descending staircases in 1 mA steps. EPT was defined as the average stimulation intensity (mA) of the last 2 peaks and 2 troughs of the last two ascending‐descending staircases.

### 2.5 Experimental Procedure

Stimulus intensity was set to 150% of EPT to ensure a painful stimulation intensity likely to evoke RIII‐reflexes during the experiment. The experiment consisted of five blocks with three trials each. Within a trial, a single pulse train was followed by a series of five pulse trains with one of three frequencies, 0.2, 1 and 2 Hz, with the latter two being within the range of frequencies that are known to evoke wind‐up (Eide 2000). Each frequency was presented only once per block and the order of frequencies within each block was randomised. In each trial, participants rated perceived pain intensity and unpleasantness of the single pulse trains and the 5th pulse train of the series, each immediately after its occurrence. We have decided to rate a single stimulus and the 5th stimulus of a series directly after the respective stimulus presentation to avoid a rating bias (which might occur if the single and the last stimulus of the series are both rated after the end of a trial). Pain ratings were assessed by a visual analogue scale (VAS), presented on a screen, with the anchors ‘not painful’ or ‘not unpleasant’ and ‘strongest pain imaginable’ or ‘very unpleasant’. Participants used the arrow keys of a keyboard to move a cursor on the screen. Participants were instructed to rate pain intensity and unpleasantness as quickly as possible after the respective stimulus presentation. However, there was no time limit for both ratings, and the protocol was only continued after both ratings was completed. The answers of the VAS scales were converted in values ranging from 0 (‘not painful’, ‘not unpleasant’) to 100 (‘strongest pain imaginable’, ‘very unpleasant’). To draw attention to the stimulation after each rating phase, participants were instructed to close their eyes and focus on the ankle of the right leg during the stimulation phase. Within one trial, the period after ratings of the single pulse train and the start of the series of pulse trains varied randomly between 1.5 and 3 s. The resting period after ratings of the 5th stimulus varied randomly between 8 and 12 s within (i.e., inter‐trial interval) and consisted of 1–2 min between blocks (i.e., inter‐block interval). The experimental protocol is depicted in Figure 1. Each participant received 90 pulse trains (15 single pulse trains and 15 series of 5 pulse trains) in the course of the experiment. Two participants with BPD terminated the experiment at a very late phase (both during the penultimate of 5 blocks) due to strong dissociation or intolerable pain, respectively. However, as most of the data of these participants were available, their data were included in the final analysis, with missing data for the last trials. The entire experiment was controlled by Presentation (v17.0; Neurobehavioral Systems Inc., Albany, CA, USA).

### 2.6 EMG Data Preprocessing

Due to technical problems during recording, EMG data of three NCC were not available. Further, there were missing data for two participants with BPD who terminated the experiment late in the experimental procedure, and some missing data for four participants with BPD and two NCC (e.g., due to system failure of the recording computer). In total, we recorded EMG responses to 2091 (97%) pulse trains from *n* = 24 participants with BPD and 1841 (85%) pulse trains from *n* = 21 NCC (in total 3932 stimuli). For offline analysis of the EMG data, we used Spike2 software (Cambridge Electronic Design Lfg, Cambridge, England, version 5.21). The EMG signal was rectified and the Spike2 built‐in DC correction (time constant 0.02 s) was applied to remove low‐frequency electric drift. A visual inspection of the DC‐corrected data of all participants revealed that EMG data of one participant with BPD were affected by a non‐physiological artefact and were therefore excluded from further analyses of EMG data. Furthermore, based on the recorded event markers, in 0.005% of the stimuli (21 out of 3932), it could not be excluded that the pulse train was presented incorrectly (pulse train consisting of fewer than 5 pulses). As an incorrect stimulation protocol could have influenced (TS) of EMG response as well as (TS) of pain perception, the trials with least one incorrect stimulus were excluded from further analysis, both of EMG data and perceptual data. Visual inspection revealed no further artefacts except for the inevitable muscle artefacts during repetitive stimulation, especially in the 2 Hz series (see below for a description of how these were addressed). We calculated the RIII‐reflex interval *z* score by applying the formula $\frac{\text{reflex interval mean}-\text{baseline mean}}{\text{baseline standard deviation}}$, resulting in a standardised EMG response score measured in standard deviation units relative to baseline. The reflex window was defined as 90‐150 ms after stimulus onset, whereas the 60 ms pre‐stimulus interval served as baseline interval (Rhudy and France 2007). Due to inevitable baseline contamination in the course of a TS series, for all pulse trains within a TS series, the baseline of the first pulse train of the respective series was used for baseline correction (Terry et al. 2011). A valid RIII‐reflex response was defined as a mean EMG response in the reflex interval that exceeded the individual mean EMG activity during the baseline interval by at least 1 SD (Rhudy et al. 2005). The advantages of using a standardised and automated procedure to determine a valid reflex are that (a) an objective criterion is used and (b) a large amount of data can be processed efficiently. A drawback of the automated procedure is the risk of misinterpreting predominant muscle artefacts as valid reflexes, especially if the baseline of the first pulse train is used for baseline correction in the TS series. In our study, this particularly affects the 5th stimulus of the 2 Hz series, as during a first visual inspection muscle artefacts were clearly more pronounced at this frequency compared to both lower frequencies and the single stimuli. For this reason, AL and RBB inspected all 5th stimuli of the 2 Hz series independently and rated whether an RIII reflex was present or not. The interrater agreement between both raters was Cohen's *κ* = 0.82 which is considered ‘almost perfect’ (Landis and Koch 1977). In a second step, the stimuli for which there was disagreement were inspected and discussed, and a consensus was reached. The agreement between the final evaluation of the two raters and the automated procedure was Cohen's *κ* = 0.74 which is considered substantial (Landis and Koch 1977). Therefore, the automated procedure can be considered valid, and all stimuli were classified as either valid reflex or non‐valid reflex based on the automated procedure. However, if not explicitly described otherwise, we decided to include all EMG responses into the analyses to capture the full picture of modulation including low modulation between two responses below the reflex threshold as well as high modulation if only one of two reflexes was above the reflex threshold.

### 2.7 Statistical Analyses

All statistical analyses were conducted in the R environment (R Core Team 2021). Besides test statistics and *p*‐values, we report absolute values of effect sizes computed as Cohen's *d* or *r*, when applicable. Data of thresholds and stimulation intensities were tested for normal distribution using the Shapiro–Wilk test. If the assumption of normality was violated, non‐parametric statistics were used.

#### 2.7.1 Thresholds and Stimulation Intensity in BPD and NCC

To test for differences in detection and pain thresholds, we compared data of participants with BPD and NCC using two‐tailed two‐sample *t*‐tests or, in the case of non‐normal distribution, with the non‐parametric equivalent, that is, Mann–Whitney *U* tests.

#### 2.7.2 Linear Mixed Effect Models

Experimental data were analysed with linear mixed effects models (LMM) using the *lmerTest* package (Kuznetsova et al. 2017) and the *lmer* function. Significance of the fixed effects was tested using the *anova* function, applying Satterthwaite's method to estimate degrees of freedom. Significant main effects and interactions were followed by pairwise post hoc comparisons of the estimated marginal means using *emmeans* (Lenth 2022). Where appropriate, correction for multiple testing was applied using Bonferroni corrections. In all our LMMs, the random effect (1| subject) allows for variable intercepts for each subject. Because of the way variance is partitioned in LMMs (e.g., Rights and Sterba 2019), there is no agreed‐on method to calculate standard effect sizes for individual model terms such as main effects or interactions. Therefore, we do not report effect sizes for main or interaction effects of LMMs. Nevertheless, we used LMMs because mixed models are superior to alternative approaches in controlling for Type 1 errors, and results from mixed models are more likely to generalise to new observations (e.g., Barr et al. 2013).

#### 2.7.3 Pain Perception and Reflex Responses to Single Stimuli in BPD and NCC

In order to test for differences in pain perception and reflex responses to the stimuli, we analysed the effect of group (NCC vs. BPD), frequency (0.2 Hz vs. 1 Hz vs. 2 Hz) and the group by frequency interaction on pain intensity and unpleasantness as well as the EMG responses related to the single pulse trains by using three separate LMMs. We further correlated EMG responses on single pulse trains with pain intensity and unpleasantness using Spearman rank correlations (*r*_s_).

#### 2.7.4 TS in BPD and NCC

We report arithmetic means and standard deviations of pain intensity, pain unpleasantness and EMG responses of the single pulse train and the 5th pulse train of a TS series for both groups and each frequency separately. In order to test for differences in TS, we analysed the effect of stimulus (single stimulus vs. 5th stimulus of a series, i.e., TS), frequency (0.2 Hz vs. 1 Hz vs. 2 Hz), group (NCC vs. BPD), and their interactions on pain intensity and pain unpleasantness as well as the EMG response by using three separate LMMs.

For the EMG response, the LMM was repeated taking only trials with valid reflexes into account. Further, to control for the effect of (different) stimulation intensities between NCC and BPD, an additional LMM on EMG responses was performed with stimulation intensity as a fixed factor in addition to stimulus, frequency and group. Both additional analyses are reported in the supplement.

In the supplement we further report arithmetic means and standard deviations of the difference scores for both groups and each frequency. These scores relate to the difference in pain intensity, pain unpleasantness and EMG response between the values of the 5th pulse train of a series and the value of the preceding single pulse train, with positive values indicating an increase and thus TS (e.g., Marouf et al. 2015).

#### 2.7.5 Correlation Between TS of Reflex Responses and TS of Pain

To assess the association between TS of the reflex response and TS of pain, we restricted the analysis to the results of the 2 Hz trials, because only these (but not 1 Hz trials) differed significantly from the EMG responses at the baseline condition of 0.2 Hz, which is in line with previous studies (Terry et al. 2011). We correlated TS of the EMG response at 2 Hz with TS of pain intensity and TS of pain unpleasantness for each group separately using Spearman rank correlations (correlation coefficient *r*_s_). We further used non‐parametric partial correlation for testing the relationship between TS of the reflex response at 2 Hz with TS of pain intensity and TS of pain unpleasantness while controlling for applied stimulus intensity.

#### 2.7.6 Association Between TS of Pain, Pain Thresholds and Clinical Markers in BPD

We correlated TS of the reflex response, TS of pain intensity and TS of pain unpleasantness at 2 Hz with clinical markers of symptom severity, state and trait dissociation, change in state dissociation (from pre‐ to post‐stimulation with positive values indicating an increase in dissociation), frequency of NSSI, state anxiety, as well as pain threshold within the BPD group.

#### 2.7.7 Supplemental Analyses

For better generalizability of the results, we did not exclude participants who (a) reported former injury (e.g., torn ligament or ankle sprain) which did not cause any scars in the stimulation area, or (b) reported regular pain or (c) reported intake of SSRI, *and* were *no* statistical outliers in our pain measurements a priori. However, in the supplement, we further report the results of those tests of the main analysis that revealed significant effects (comparison of pain threshold between groups, LMMs on pain intensity, pain unpleasantness and EMG response as well as correlation between TS of pain and TS of reflex response), after excluding data of these participants.

## 3 Results

### 3.1 Thresholds and Stimulation Intensity in BPD and NCC

Descriptive statistics for detection and pain thresholds as well as stimulation intensity can be found in Table 2. There was no significant difference in detection threshold between BPD and NCC, *z* = −1.25, *p* = 0.21, *r* = 0.18. Because the stimulation was a multiple of the pain threshold, both the pain threshold and the stimulation intensity differed significantly between both groups *t*_46_ = −3.74, *p* < 0.001, *d* = 1.08, with the BPD group having higher values.

**TABLE 2:** Perception and pain thresholds as well as stimulation intensities in participants with borderline personality disorder and non‐clinical controls.

	Detection threshold (mA)			Pain threshold (mA)			Stimulation intensity (mA)		
	BPD (*n* = 24)	NCC (*n* = 24)	Group comparison (*p*)	BPD (*n* = 24)	NCC (*n* = 24)	Group comparison (*p*)	BPD (*n* = 24)	NCC (*n* = 24)	Group comparison (*p*)
*M* ± SD	0.81 ± 0.46	0.62 ± 0.29	0.021	6.97 ± 2.45	4.53 ± 2.06	< 0.001***	10.46 ± 3.67	6.79 ± 3.10	< 0.001***
Mdn; IQR	0.50; 1.00	0.50; 0.00		6.38; 3.06	3.88; 3.51		9.6; 4.60	5.81; 5.27	

Abbreviations: BPD, participants with borderline personality disorder; IQR, interquartile range; *M*, mean; Mdn, median; *n*, number; NCC, non‐clinical controls; SD, standard deviation.

*** *p* < 0.001.

### 3.2 Pain Perception and EMG Responses to Single Stimuli in BPD and NCC

We observed no significant effect of group, frequency, or group*frequency interaction on pain intensity (all *F* < 0.28, all *p* > 0.67), pain unpleasantness (all *F* < 0.81, all *p* > 0.44), or EMG responses (all *F* < 1.89, all *p* > 0.18) of the single stimuli. By taking only the EMG responses of valid RIII‐reflexes into account (see Table S2 for number of valid reflexes per group), the resulting pattern of the LMM on EMG responses of the single stimuli remained unaltered (all *F* < 0.48, all *p* > 0.61).

There was no significant correlation between EMG‐responses and pain intensity or unpleasantness, neither in BPD (all *r*_s_ < 0.6, all *p* > 0.77) nor in NCC (all *r*_s_ < 0.28, all *p* > 0.21). This was also true if only valid RIII‐reflexes and the respective ratings were analysed (all *r*_s_ < 0.12, all *p* > 0.78).

### 3.3 TS in BPD and NCC

Descriptive data for pain intensity and pain unpleasantness as well as EMG responses for the single and the 5th pulse train of a TS series in different stimulation frequencies can be found in Table 3. In the supplement, we further report and visualise descriptive data for TS of pain intensity, TS of pain unpleasantness and TS of EMG response, based on the difference scores of the single and the 5th pulse train of a TS series (see Table S1 and Figure S1).

**TABLE 3:** Pain intensity, pain unpleasantness, and reflex response for the single pulse train and 5th pulse train of a temporal summation series in participants with borderline personality disorder and non‐clinical controls.

Frequency	BPD (*n* = 24)						NCC (*n* = 24)					
	0.2 Hz		1 Hz		2 Hz		0.2 Hz		1 Hz		2 Hz	
	Single	5th	Single	5th	Single	5th	Single	5th	Single	5th	Single	5th
	*M* (SD)	*M* (SD)	*M* (SD)	*M* (SD)	*M* (SD)	*M* (SD)	*M* (SD)	*M* (SD)	*M* (SD)	*M* (SD)	*M* (SD)	*M* (SD)
Intensity (VAS 0–100)	36.73 (23.39)	40.95 (23.86)	36.68 (24.25)	51.10 (23.42)	35.33 (23.79)	54.74 (23.45)	33.57 (24.99)	40.11 (26.25)	34.37 (24.49)	47.43 (26.54)	33.07 (23.96)	53.97 (26.45)
Unpleasantness (VAS 0–100)	40.75 (23.68)	48.70 (22.73)	40.64 (24.32)	60.75 (20.67)	39.34 (23.81)	66.08 (21.82)	39.35 (25.04)	45.28 (25.98)	41.34 (24.60)	54.32 (26.51)	39.13 (23.12)	61.68 (27.62)
Reflex response (standardised score)	1.87 (3.40)	1.66 (2.83)	2.06 (4.35)	2.55 (3.32)	1.84 (2.95)	4.01 (6.71)	0.63^a^ (1.67)	0.69^a^ (2.07)	1.04^a^ (2.66)	1.76^a^ (4.93)	0.97^a^ (2.57)	3.31^a^ (5.34)

Abbreviations: BPD, participants with borderline personality disorder; *M*, mean; *n*, number; NCC, non‐clinical controls; SD, standard deviation; VAS, visual analogue scale.

^a^ *n* = 21.

**FIGURE 2:**
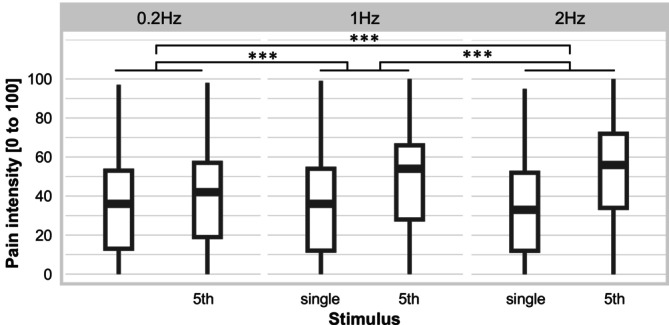
Ratings of pain intensity for single pulse trains and 5th pulse trains of a temporal summation sequence at three stimulation frequencies and for both groups (individuals with and without borderline personality disorder) together. Boxplots: Medians and quartiles are marked by the lines of the boxes. Whiskers indicate 1.5 inter‐quartile range or minimum/maximum value. ***p < 0.001.

For pain intensity, there was a significant main effect of stimulus with higher pain intensity reported for the 5th stimulus compared to the single stimulus (i.e., TS of pain intensity), *F*_1,1322_._03_ = 380.98, *p* < 0.001. There was also a significant main effect of frequency, *F*_2,1322_._14_ = 35.02, *p* < 0.001, as well as a significant stimulus*frequency interaction, *F*_2,1322_._03_ = 40.94, *p* < 0.001. All post hoc tests were significant with positive estimates, indicating that—independent of the group—the effect of stimulus on pain intensity (i.e., TS of pain intensity) was significantly stronger for 2 Hz compared to 1 and 0.2 Hz stimulation, and also for 1 Hz compared to 0.2 Hz (see Table 4 and Figure 2). However, neither the main effect of group nor any of the interactions with group were significant (all *F* < 0.69, all *p* > 0.50), that is, there were no significant differences in TS of pain intensity between groups.

**TABLE 4:** Results of post hoc pairwise comparisons of linear mixed models for pain intensity and unpleasantness as well as reflex responses.

		Estimate	SE	df	*t*	*p*_Bonf_
Pairwise comparisons of the stimulus by frequency interaction for perceived pain intensity						
Single stimulus vs. 5th stimulus of series	0.2 Hz vs. 1 Hz	8.36	1.64	1322	5.09	< 0.001
	0.2 Hz vs. 2 Hz	14.78	1.65	1322	8.97	< 0.001
	1 Hz vs. 2 Hz	6.42	1.64	1322	3.91	< 0.001
Pairwise comparisons of the stimulus by frequency interaction for perceived pain unpleasantness						
Single stimulus vs. 5th stimulus of series	0.2 Hz vs. 1 Hz	9.61	1.61	1322	5.98	< 0.001
	0.2 Hz vs. 2 Hz	17.70	1.61	1322	11.00	< 0.001
	1 Hz vs. 2 Hz	8.10	1.60	1322	5.05	< 0.001
Pairwise comparisons of the stimulus by group interaction for perceived pain unpleasantness						
Single stimulus vs. 5th stimulus of series	NCC vs. BPD	4.45	1.31	1322	3.39	< 0.001
Pairwise comparisons of stimulus by frequency interaction for reflex response						
Single stimulus vs. 5th stimulus of series	0.2 Hz vs. 1 Hz	0.67	0.43	1182	1.55	0.41
	0.2 Hz vs. 2 Hz	2.82	0.43	1182	6.46	< 0.001
	1 Hz vs. 2 Hz	2.15	0.43	1182	4.95	< 0.001

Abbreviations: BPD, participants with borderline personality disorder; df, degrees of freedom; Hz, hertz; NCC, non‐clinical controls; SE, standard error.

**FIGURE 3:**
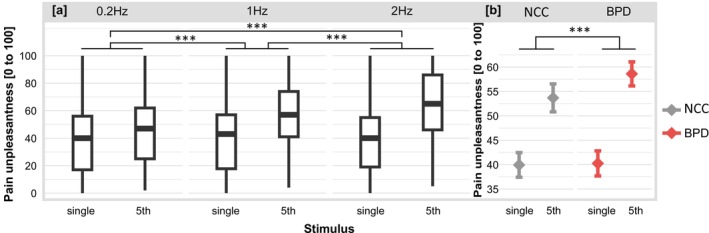
Ratings of pain unpleasantness for the single pulse trains and 5th pulse trains of a temporal summation sequence for both groups, participants with borderline personality disorder (BPD) and non‐clinical controls (NCC). (a) Shows ratings for both groups together separately for three stimulation frequencies. Boxplots: Medians and quartiles are marked by the lines of the boxes. Whiskers indicate 1.5 inter‐quartile range or minimum/maximum value. (b) Shows means and 95% CIs of the ratings for all three stimulation frequencies together, separately for BPD and NCC. ***p < 0.001.

For pain unpleasantness, there was a significant main effect of stimulus with higher pain unpleasantness reported for the 5th stimulus compared to the single stimulus (i.e., TS of pain unpleasantness), *F*_1,1322_._03_ = 598.21, *p* < 0.001. There was also a significant main effect of frequency, *F*_2,1322_._14_ = 57.60, *p* < 0.001, as well as a significant stimulus*frequency interaction *F*_2,1322_._03_ = 60.58, *p* < 0.001. All post hoc tests were significant with positive estimates, indicating that—independent of the group—the effect of the stimulus on pain unpleasantness (i.e., TS of pain unpleasantness) was significantly stronger for 2 Hz compared to 1 Hz and 0.2 Hz, and for 1 Hz compared to 0.2 Hz (see Table 4 and Figure 3a). Furthermore, for pain unpleasantness, there was a significant group*stimulus interaction *F*_1,1322_._03_ = 11.50, *p* < 0.001. A post hoc test of this interaction was significant with a positive estimate (see Table 4), indicating that—independent of the frequency—the effect of stimulus on pain unpleasantness (i.e., TS of pain unpleasantness) was significantly stronger in BPD compared to NCC (see Figure 3b). There was no significant main effect of group or group*frequency interaction or group*frequency*stimulus interaction (all *F* < 1.29, all *p* > 0.28).

**FIGURE 4:**
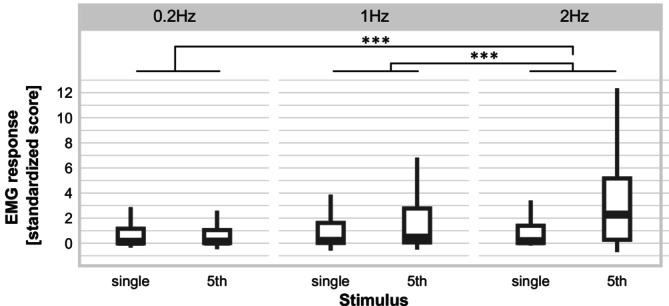
EMG responses for single pulse trains and 5th pulse trains of a temporal summation sequence at three stimulation frequencies and for both groups (individuals with and without borderline personality disorder) together. Boxplots: Medians and quartiles are marked by the lines of the boxes. Whiskers indicate 1.5 inter‐quartile range or minimum/maximum value. ***p < 0.001.

For the EMG response, there was a significant main effect of stimulus with a higher EMG response for the 5th stimulus compared to the single stimulus (i.e., TS of EMG response) *F*_1,1182_._22_ = 37.87, *p* < 0.001. There was also a significant main effect of frequency *F*_2,1182_._73_ = 28.45, *p* < 0.001 as well as a significant stimulus*frequency interaction *F*_2,1182_._22_ = 22.80, *p* < 0.001. Post hoc pairwise comparisons revealed that the effect of stimulus on EMG response (i.e., TS of EMG response) was stronger at 2 Hz compared to 1 Hz and 0.2 Hz, but no significant difference between 0.2 Hz and 1 Hz emerged (see Table 4 and Figure 4). The main effect of group or the interactions with group were not significant (all *F* < 2.51, all *p* > 0.12), that is, there were no significant differences in TS of the EMG response between groups.

Results for EMG responses did not significantly change after controlling for the absolute level of stimulation (see Table S4). When only those trials with at least one valid RIII‐reflex were considered (see Table S2 for number of valid reflexes per group), there was still a significant main effect of frequency, as well as a trend for significance for a main effect of stimulus or stimulus*frequency (see Table S3).

### 3.4 Correlation Between TS of Pain and TS of EMG Responses

**FIGURE 5:**
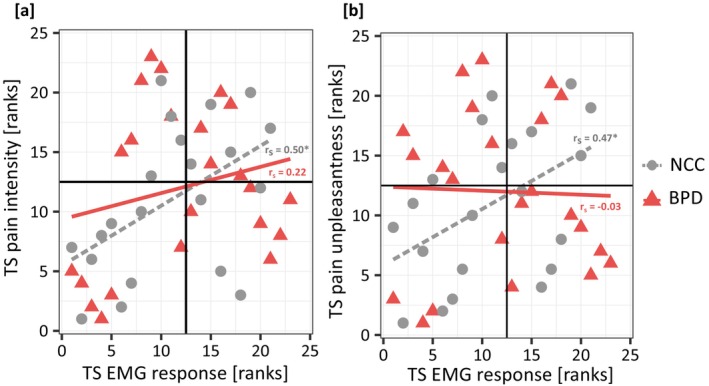
Association (spearman correlation *r*_s_) between temporal summation of reflex response and perceived pain intensity (a) and pain unpleasantness (b) in participants with borderline personality disorder (BPD) and non‐clinical controls (NCC); EMG, electromyogram; TS, temporal summation. *p < 0.05.

For NCC, there was a significant positive correlation between TS of the EMG response and TS of pain intensity (*r*_s_ = 0.50, *p* = 0.02) and TS of pain unpleasantness (*r*_s_ = 0.47, *p* = 0.03). In BPD, however, TS of the EMG response was not significantly associated with TS of pain intensity or TS of pain unpleasantness (all *r*_s_ < 0.22, all *p* > 0.31) (see Figure 5 and Table 5).

**TABLE 5:** Association between temporal summation of pain, pain threshold, and clinical markers in participants with borderline personality disorder.

	Trait dissociation (FDS)	Symptom severity (BSL‐23)	Frequency NSSI last month	State anxiety (STAI)	State dissociation pre (DSS‐4)	Change in state dissociation (post–pre)	TS pain intensity	TS pain unpleasantness	Pain threshold
TS EMG response	*r*_s_ = 0.31, *p* = 0.15	*r*_s_ = 0.28, *p* = 0.20	*r*_s_ = 0.21, *p* = 0.36	*r*_s_ = 0.13, *p* = 0.56	*r*_s_ = 0.03, *p* = 0.99	*r*_s_ = −0.33, *p* = 0.14	*r*_s_ = 0.22, *p* = 0.31	*r*_s_ = −0.03, *p* = 0.88	*r*_s_ = 0.15, *p* = 0.50
TS pain intensity	*r* = −0.22, *p* = 0.32	*r* = −0.06, *p* = 0.79	*r*_s_ = 0.02, *p* = 0.93	*r* = −0.09, *p* = 0.66	*r*_s_ = 0.12, *p* = 0.58	*r*_s_ = −0.35, *p* = 0.10		***r* = 0.77, *p* < 0.001** *	*r* = 0.26, *p* = 0.22
TS pain unpleasantness	*r* = −0.06, *p* = 0.77	*r* = −0.18, *p* = 0.39	*r*_s_ = −0.15, *p* = 0.51	*r* = −0.04, *p* = 0.84	*r*_s_ = 0.20, *p* = 0.34	*r*_s_ = −0.38, *p* = 0.07			*r* = 0.36, *p* = 0.08
Pain threshold	*r* < −0.01, *p* = 0.98	*r* = 0.23, *p* = 0.30	*r*_s_ = 0.10, *p* = 0.66	*r* = 0.07, *p* = 0.72	*r*_s_ = 0.05, *p* = 0.83	*r*_s_ = −0.23, *p* = 0.28			

*Note:* Uncorrected *p*‐values are reported. Bold font indicates significant results.

Abbreviations: BSL‐23, Borderline Symptom List (Bohus et al. 2009); DSS‐4, Dissociation‐Tension Scale acute (short version) (Stiglmayr et al. 2009); FDS, Fragebogen zu Dissoziativen Symptomen [Questionnaire of dissociative symptoms] (Freyberger et al. 1999) German version of the Dissociative Experience Scale (Bernstein and Putnam 1986); NSSI, non‐suicidal self‐injury; post, after the stimulation; change in state dissociation was assessed as difference score with positive values indicating an increase in dissociation from pre‐ to post‐stimulation; pre, before the stimulation started; STAI, Stait–Trait Anxiety Inventory (Laux et al. 1981); TS, temporal summation.

* Results that survived Bonferroni correction (*p*_Bonf_ < 0.05).

Results of the partial correlation controlling for applied stimulus intensity revealed that the correlations in NCC were no longer significant; there only was a trend for a positive relationship between TS of the EMG response and TS of pain intensity (*r*_s_ = 0.37, *p* = 0.10) and TS of pain unpleasantness (*r*_s_ = 0.43, *p* = 0.06). In BPD, the relationship between TS of the EMG response and TS of pain intensity and TS of pain unpleasantness remained non‐significant (all *r*_s_ < 0.15, all *p* > 0.49).

### 3.5 Association Between TS of Pain, Pain Thresholds and Clinical Markers in BPD

There was a trend towards significance for a negative association between change in state dissociation from pre‐ to post‐stimulation with TS of pain intensity (*r*_s_ = −0.35, *p* = 0.10) and TS of pain unpleasantness (*r*_s_ = −0.38, *p* = 0.07). None of the other correlations between TS of the EMG response, TS of pain intensity, or TS of pain unpleasantness with the assessed clinical markers was significant. There was also no significant correlation between TS of pain or TS of EMG response with pain threshold in BPD (see Table 5).

Result patterns of the main analysis, in terms of significant/non‐significant findings, after excluding subjects who (a) reported former injury in the stimulation area, or (b) reported regular pain or (c) reported intake of SSRI did not differ from the results of the entire sample (see Tables S5–S7).

## 4 Discussion

In this study, we investigated pain processing in participants with BPD compared to NCC. Using electric stimulation, we assessed TS of pain and TS of the RIII‐reflex to three different frequencies of painful stimulation as well as pain thresholds. We related TS of pain and TS of the RIII‐reflex to each other and examined the relationship of pain measures and clinical markers of BPD.

We replicated previous findings of enhanced electrical pain thresholds in BPD (e.g., Ludäscher et al. 2007), which is in line with generally reduced pain sensitivity in BPD (Fales et al. 2021). Interestingly, there was no significant difference between BPD and NCC in the RIII‐reflex to single stimuli that were adjusted to the individual pain threshold, suggesting comparable spinal activity for perceptually comparable painful stimulation in both groups. In line with previous results (Defrin et al. 2020; Ginzburg et al. 2018), there was no significant difference in TS of pain intensity between both groups. However, TS of pain unpleasantness was significantly higher in BPD compared to NCC, independent of stimulation frequency. Our main result identified enhanced TS of pain unpleasantness as a feature of BPD, which seems surprising in the context of meta‐analytic evidence for reduced pain perception, in terms of increased pain thresholds in BPD (Fales et al. 2021). However, although static pain measures such as pain thresholds are indicative of the basic state of the nociceptive system, temporal summation of pain is a dynamic pain measure activating a specific pain mechanism (Arendt‐Nielsen and Yarnitsky 2009). It has been shown that, even within one modality, static and dynamic pain measures are not strongly interrelated, indicating that they represent distinct factors of pain perception (Hastie et al. 2005). Based on our current results, we suggest that altered pain perception in BPD is composed of alterations in several pain processes that go beyond mere pain insensitivity and even involve pain amplification.

Interestingly, increased prevalence of chronic pain has been described in BPD, and its discrepancy with acute pain insensitivity has been termed ‘the pain paradox’ (Sansone and Sansone 2007). In a sample of participants with chronic pain, it has been shown that BPD symptoms were associated with enhanced affective pain experiences and polysomatic complaints associated with central sensitization, that is, increased responsiveness of the central nervous system (Johnson et al. 2020). Even if central sensitization and wind‐up/TS of pain are not equivalent (Woolf 1996), it has been shown that neuronal events leading to wind‐up also trigger an expansion of receptive fields of dorsal horn neurons and increase responses to C‐fibre stimulation, representing classical characteristics of central sensitization, suggesting a shared mechanism (Li et al. 1999). Both, an animal study on sensitised dorsal horn neurons resulting from the repetitive exposure to stressful events (Hoheisel et al. 2015) and a human study in participants with chronic pain found an association between increased wind‐up and early‐life stress (Tesarz et al. 2016), suggesting that this kind of stress, which is a predictor for BPD (Ball and Links 2009), might result in a hyperexcitability of the central somatosensory system. An experimental investigation of the pain paradox in a student sample including individuals with and without a history of NSSI revealed that the paradoxical occurrence of reduced acute pain and increased clinical pain was specific to participants with BPD features and a history of NSSI (Carpenter and Trull 2015). Together with our current findings, these results suggest that frequent nociceptive input, in terms of NSSI, combined with a hyperexcitability of the central nervous system might result in increased TS of pain unpleasantness, central sensitization and related chronic pain states in BPD. However, our current results did not provide evidence for a significant difference in TS of the RIII‐reflex between BPD and NCC.

We could replicate previous findings of a significant positive correlation between TS of the RIII‐reflex and TS of pain intensity in NCC (c.f., Marouf et al. 2015) and extended it by showing that TS of pain unpleasantness was also significantly positively correlated with TS of the RIII‐reflex in NCC. This suggests that in NCC both the sensory *and* affective components of TS of pain are significantly related to spinal nociceptive processing. However, the results of the partial correlation, controlling for applied stimulus intensity, in NCC suggest that the significant association between TS at the reflex and perceptual levels of pain in NCC is largely explained by their mutual dependence on stimulus intensity. In contrast, our results revealed no significant evidence of an association between TS of pain and TS of the RIII‐reflex in BPD (regardless of whether it was controlled for stimulus intensity or not).

In contrast to previous results in BPD (Defrin et al. 2020), in our study TS of pain intensity was not significantly correlated with pain threshold, and there was no significant correlation between TS of pain unpleasantness and pain threshold. This might indicate that different mechanisms underlie enhanced TS of pain unpleasantness and reduced pain sensitivity in BPD. In contrast to our repetitive stimulation protocol, Defrin et al. (2020) used tonic heat pain, which has been shown to evoke TS of pain before (Granot et al. 2006; Kleinböhl et al. 1999). These divergent results of our current study and previous results (Defrin et al. 2020) complement the findings of a study comparing TS of pain evoked by tonic and repetitive stimuli revealing that although both types of summation are positively correlated, only tonic TS of pain was significantly negatively associated with pain thresholds (Granot et al. 2006). Specifically, heightened repetitive TS of pain has been related to state anxiety assessed immediately before the experiment, presumably because fear of pain increased pain perception along the repetitive stimuli (Granot et al. 2006). In contrast, in the current study, state anxiety was not related to TS of pain intensity or TS of pain unpleasantness. However, anxiety, which is a common feature in BPD (Bohus et al. 2021), was assessed on a separate day in our study and was therefore likely to be less influenced by fear of pain. Interestingly, anxiety, and especially pain‐related anxiety, has been found to account for the association between BPD features and pain perception of clinical pain in participants with chronic pain (Reynolds et al. 2018). The same study found that affective lability plays an important role in the association between BPD features and clinical pain perception. The authors concluded that emotional reactivity to pain might substantially contribute to enhanced chronic pain complaints in BPD individuals (Reynolds et al. 2018). This conclusion is partly supported by a study using momentary assessment methods, which reported pain‐related behavioural dysregulations in BPD in terms of increased and more variable everyday pain as well as enhanced negative affect in response to everyday pain (Carpenter et al. 2019). Increased TS of pain unpleasantness might thus constitute a marker for pain‐related affective dysregulation in BPD; however, its association with chronic pain needs to be assessed in future studies.

For the TS protocol, the effect of stimulus on TS of pain was significantly higher at 2 Hz vs. 1 Hz vs. 0.2 Hz stimulation, independent of the group. This is in line with previous results, indicating stronger TS of pain at higher stimulation frequencies in NCC (e.g., Kleinböhl et al. 2006). Independent of the group, TS of the RIII‐reflex did not significantly differ between 1 and 0.2 Hz stimulation, but both differed from 2 Hz stimulation, supporting previous results that TS of the RIII‐reflex is most pronounced with 2 Hz stimulation in NCC (Terry et al. 2011). In general, this supports the validity of our experimental protocol, strengthening our results.

### 4.1 Limitations and Future Directions

Legs were covered with a blanket during EMG recording, providing an additional (non‐painful) sensory stimulation, which might have influenced signal processing. Additionally, we adjusted stimulation intensity to the individual pain threshold, which may be below the RIII‐reflex threshold. This might have resulted in stimulation intensities not high enough to reliably evoke RIII‐reflexes, limiting the interpretability of the results on the reflex responses. This might be true especially for NCC, as the mean stimulation intensity in this group (6.79 mA) was below the reported RIII‐reflex threshold for NCC (8.6–10.8 mA) (Skljarevski and Ramadan 2002). Particularly, EMG response strength to the single stimuli was small in NCC (see Table 3) and the number of valid reflexes was low in both groups, especially in response to the single stimuli (20% for NCC and 39% for BPD). Consequently, a firm conclusion regarding spinal activity, especially in response to the single stimulus, is not possible. However, the number of valid reflexes was higher in response to the 5th stimulus of a series, especially in the 2 Hz condition (49% for NCC and 76% for BPD) and the main result patterns were comparable when only trials with at least one valid RIII‐reflex were considered or after controlling for the effect of stimulus intensity. Future studies are necessary to disentangle altered nociceptive processing on spinal level and pain perception in BPD, as well as its association with peripheral nociceptive input, by applying stimulation intensities based also on RIII‐reflex thresholds, as well as standardised stimulation intensities. These studies might further consider applying more conservative baseline corrections in TS protocols (e.g., correction for local baseline) (Terry et al. 2011) or measuring the TS reflex threshold.

Our sample size was relatively small and consisted solely of females. There is a sex effect on TS of pain, with women showing enhanced TS of pain compared to men, indicating sex‐specific differences in central processing of nociceptive stimuli (Sarlani et al. 2004). Whether our results on altered TS of pain can be generalised to male participants with BPD needs to be investigated in future studies. Replication of our results in larger studies might be interesting, especially for validating the weak associations between TS of pain and changes in dissociation from pre‐ to post‐stimulation, which showed a small, only marginally significant effect (*r*_s_ = −0.41; *p* = 0.054) in the present study. Future studies in larger samples should also investigate other pain modalities (thermal or mechanical stimuli) to check whether the results can be generalised across different pain modalities.

Another limitation is that intake of SSRIs was not interrupted for study participation. SSRIs have been successfully used to treat chronic pain (Patetsos and Horjales‐Araujo 2016) and might thus have influenced our results. Furthermore, the inclusion of a few subjects who reported regular pain episodes or former injury in the stimulation area might have influenced the results. However, the pattern of results remained comparable with and without these participants. Future studies need to compare BPD participants with and without chronic pain to further investigate paradoxical pain perceptions and the association between increased TS of pain unpleasantness and chronic pain. Future studies on pain in BPD should not only assess pain thresholds or ratings of painful stimuli but include measures of reflex level of pain, pain modulation and pharmacological mediators of pain modulation to disentangle mechanisms behind altered pain perception in BPD.

## Author Contributions

A.L., R.B.‐B. and H.F. designed the study. A.L., D.K., R.B.‐B. and H.F. contributed to the experimental setup. S.S. was involved in the assessment of the participants. A.L. collected the experimental data and analysed the data. A.L. drafted the manuscript. D.K., S.S., S.C.H., U.H., R.B.‐B. and H.F. provided substantive feedback and revisions of the manuscript. All authors have approved the final version of the manuscript and agree to be accountable for all aspects of the work.

## Disclosure

Study and analysis preregistration: This research was not preregistered in an independent institutional registry.

## Conflicts of Interest

The authors declare no conflicts of interest.

## Supporting information


Data S1.

## Data Availability

The data supporting the findings of this study are available from the corresponding author upon reasonable request.
