# Supplementary material for: Temporal Summation of Pain Unpleasantness Is Increased in Borderline Personality Disorder
Source: Eur J Pain. 2025 May 22;29(6):e70042. doi: 10.1002/ejp.70042 (PMC12097198; doi:10.1002/ejp.70042)
Supplement: Supplementary file 1 — Data S1. [file EJP-29-0-s001.docx]

SUPPLEMENT DIGITAL CONTENT:

**Temporal summation of pain unpleasantness is increased in borderline personality disorder**

Annette Löffler^a,b,c,d^, Dieter Kleinböhl^a^, Sylvia Steinmann^b^, Sabine C. Herpertz^e^, Ute Habel^c^, Robin Bekrater-Bodmann^a,b,c,d*^, & Herta Flor^a*^

^a^ Institute of Cognitive and Clinical Neuroscience, Central Institute of Mental Health, Medical Faculty Mannheim, Heidelberg University, Mannheim, Germany

^b^ Department of Psychosomatic Medicine and Psychotherapy, Central Institute of Mental Health, Medical Faculty Mannheim, Heidelberg University, Mannheim, Germany

^c^ Department of Psychiatry, Psychotherapy and Psychosomatics, Uniklinikum RWTH Aachen, Aachen, Germany

^d^ Scientific Center for Neuropathic Pain Aachen SCN^AACHEN^, Uniklinikum RWTH Aachen, Aachen, Germany

^e^ Department of General Psychiatry, Medical Faculty, Center for Psychosocial Medicine, Heidelberg University, Heidelberg, Germany

* Contributed equally

**Corresponding author:** Annette Löffler, Department of Psychiatry, Psychotherapy and Psychosomatics, Uniklinikum RWTH Aachen, Pauwelsstraße 30, 52074 Aachen, Germany. Tel.: +49 241 9979 3213. E-mail address: aleffler@ukaachen.de

## Additional table and figure: Descriptive statistics for temporal summation

| ***Table S1:*** Temporal summation of pain intensity, pain unpleasantness, and reflex response in participants with borderline personality disorder and non-clinical controls. | | | | | | | |
| --- | --- | --- | --- | --- | --- | --- | --- |
|  | **BPD** [*n* = 24] | | |  | **NCC** [*n* = 24] | | |
| **Frequency** | **0.2 Hz** | **1 Hz** | **2 Hz** |  | **0.2 Hz** | **1 Hz** | **2 Hz** |
|  | *M (SD)* | *M (SD)* | *M (SD)* |  | *M (SD)* | *M (SD)* | *M (SD)* |
| **TS Intensity** [VAS 0 – 100] | 4.71 (9.01) | 15.35 (13.79) | 19.66 (10.26) |  | 6.54 (8.34) | 13.05 (8.90) | 20.87 (13.83) |
| **TS Unpleasantness** [VAS 0 – 100] | 8.54 (10.30) | 20.79 (14.34) | 27.16 (13.66) |  | 5.93 (7.51) | 12.97 (5.50) | 22.49 (11.51) |
| **TS EMG responses** [standardized score] | -0.17 (1.37) | 0.57 (2.97) | 3.66 (5.53) |  | 0.06^1^ (0.18) | 0.70^1^ (3.05) | 2.50^1^ (4.91) |

BPD = borderline personality disorder; NCC = non-clinical controls; *n* = number; *M* = mean; *SD* = standard deviation; TS = temporal summation; VAS = visual analogue scale; ^1^ *n* = 21

##
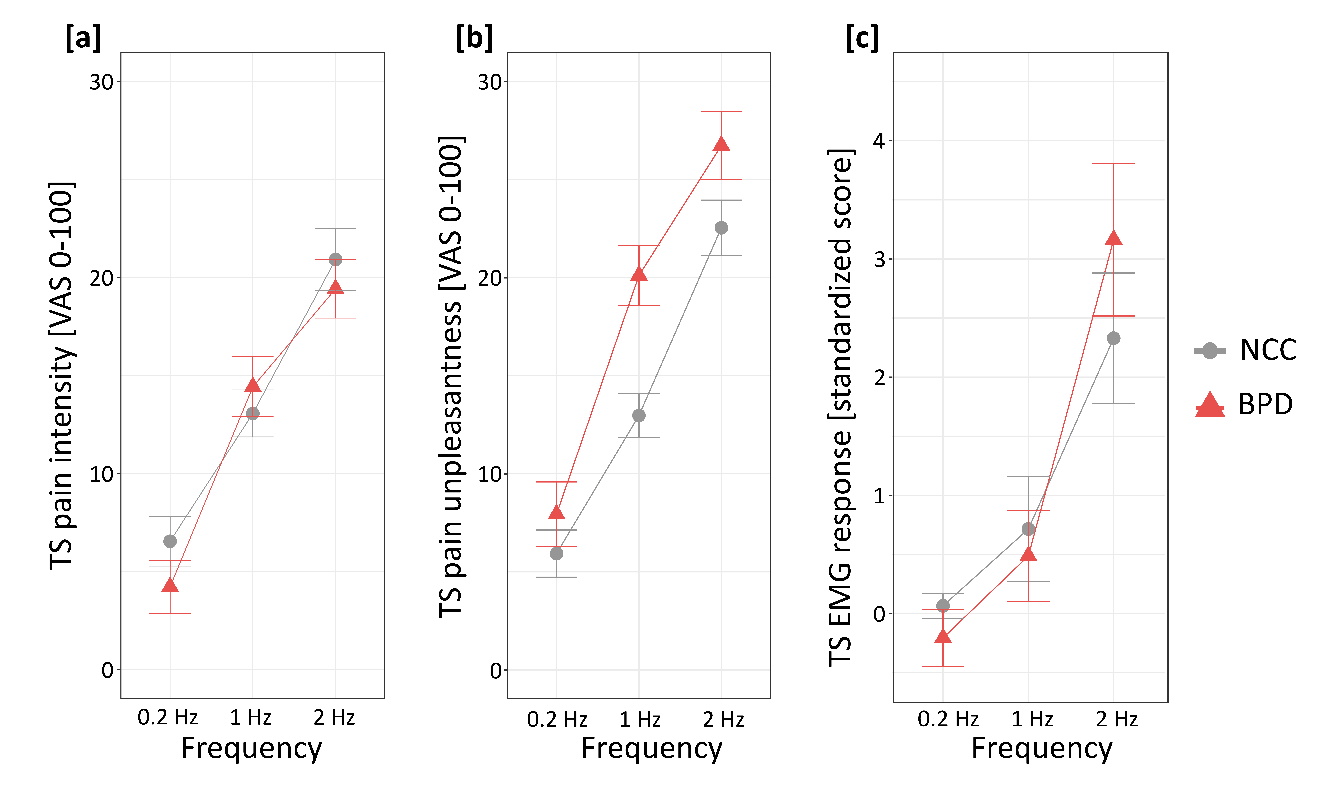


**Figure S1:** Parameters of temporal summation at three stimulation frequencies and for both groups, participants with borderline personality disorder (BPD) and non-clinical controls (NCC). **[a]** shows results of temporal summation (TS) of pain intensity ratings, **[b]** shows temporal summation of pain unpleasantness ratings, **[c]** shows temporal summation of reflex responses. Each of the three parameters is calculated as the difference between the responses to the single pulse train and the 5^th^ pulse train in the sequence of one trial; VAS = visual analog scale, EMG = electromyography.

## Additional Analysis: Linear mixed effect models (LMM) on reflex response

1. **Number of valid reflexes per group and type of stimulus**

***Table S2:*** *Number of valid reflexes per group*

| **Stimulus type** | **BPD** | | **NCC** | |
| --- | --- | --- | --- | --- |
|  | **participants**  *n* | **valid reflexes**  *n (%)* | **participants**  *n* | **valid reflexes**  *n (%)* |
| Single stimulus | 16 | 122 (39) | 9 | 61 (20) |
| 5^th^ stimulus of 0.2Hz series | 14 | 36 (35) | 5 | 18 (18) |
| 5^th^ stimulus of 1Hz series | 15 | 54 (51) | 8 | 30 (29) |
| 5^th^ stimulus of 2 Hz series | 21 | 80 (76) | 13 | 48 (49) |

BPD = borderline personality disorder; NCC = non-clinical controls; *n* = number;

1. **Effect of group (NCC vs. BPD), stimulus (single pulse train vs. 5^th^ pulse train of a series), frequency (0.2Hz vs. 1Hz vs. 2Hz), and their interaction on reflex responses. Only trials with at least one valid reflex are taken into account.**

| ***Table S3:*** *ANOVAS of linear mixed effect models for reflex response (only valid reflexes).* | | | | |
| --- | --- | --- | --- | --- |
| **Predictor** | ***df num*** | ***df den*** | ***F*** | ***p*** |
| group | 1 | 15.89 | 0.02 | 0.90 |
| stimulus | 1 | 280.68 | 3.50 | 0.06 |
| Frequency | 2 | 289.83 | 3.06 | **0.04** |
| group*stimulus | 1 | 280.68 | 0.29 | 0.59 |
| group*frequency | 2 | 289.83 | 1.12 | 0.33 |
| stimulus*frequency | 2 | 280.68 | 2.24 | 0.10 |
| group*stimulus*frequency | 2 | 280.68 | 1.25 | 0.29 |
| Statterwaite’s method was used to estimate degree of freedoms (df), *F* and *p* values, as implemented in the *R* package *lmerTest*; ANOVA = analysis of variance | | | | |

1. **Effect of group (NCC vs. BPD), frequency (0.2Hz vs. 1Hz vs. 2Hz), stimulus (single pulse train vs. 5^th^ pulse train of a series), and their interaction on reflex responses. Absolute level of stimulation intensity was taken into account as additional fixed factor to control for the effect of stimulation intensity**

| ***Table S4:*** *ANOVAS of linear mixed effect models for reflex response* | | | | |
| --- | --- | --- | --- | --- |
| **Predictor** | ***df num*** | ***df den*** | ***F*** | ***p*** |
| group | 1 | 41.14 | 0.38 | 0.51 |
| stimulus | 1 | 1182.22 | 37.87 | **<.001** |
| frequency | 2 | 1182.73 | 28.48 | **<.001** |
| stimulation intensity | 1 | 41.01 | 2.27 | 0.14 |
| group*stimulus | 1 | 1182.22 | 0.10 | 0.75 |
| group*frequency | 2 | 1182.70 | 0.54 | 0.58 |
| stimulus*frequency | 2 | 1182.22 | 22.80 | **<.001** |
| group*stimulus*frequency | 2 | 1182.22 | 1.03 | 0.36 |
| Statterwaite’s method was used to estimate degree of freedoms (df), *F* and *p* values, as implemented in the *R* package *lmerTest*; ANOVA = analysis of variance | | | | |

## Additional Analysis: Main analysis for subsample

We excluded participants who (a) reported regular pain (1 BPD and 1 NCC; back pain or migraine), (b) reported intake of SSRI (3 BPD), or (c) reported former pain episodes or injuries (e.g., torn ligament or ankle sprain) in the stimulation area (5 BPD and 3 NCC) and report results of the main analysis, which revealed significant results for the entire sample. One NCC reported regular pain and former injury. One participant with BPD reported intake of SSRI and former injury. In total n = 11 participants were excluded (8 BPD and 3 NCC), resulting in a sample size of n = 16 BPD and n = 21 NCC (n = 16 BPD and n = 18 NCC for data on reflex responses)

1. **Pain threshold**

| ***Table S5:*** Pain thresholds in participants with borderline personality disorder and non-clinical controls. | | | |
| --- | --- | --- | --- |
|  | **Pain threshold**  [mA] | |  |
|  | **BPD** (*n* = 16) | **NCC** (*n* = 11) | **Test statistic** |
| ***M* ± *SD*** | 7.09 ± 2.61 | 4.79 ± 2.06 | *t*_35_ = -3.00, *p* < 0.01, *d* = 0.98 |
| ***Mdn* (*IQR*)** | 6.75 (2.50) | 4.50 (3.55) |  |
| BPD = borderline personality disorder; NCC = non-clinical controls; *n* = number; *M* = mean; *SD* = standard deviation; *Mdn* = median; *IQR* = interquartile range. | | | |

1. **Linear mixed effect models on pain intensity, pain unpleasantness and reflex response**

| ***Table S6:*** *ANOVAS of linear mixed effect models for pain intensity, pain unpleasantness and reflex responses* | | | | | |
| --- | --- | --- | --- | --- | --- |
| **Outcome** | **Predictor** | ***df num*** | ***df den*** | ***F*** | ***p*** |
| Pain intensity | group | 1 | 35.03 | 0.11 | 0.74 |
|  | stimulus | 1 | 1019.02 | 279.46 | **< 0.001** |
|  | frequency | 2 | 1019.13 | 26.02 | **< 0.001** |
|  | group*stimulus | 1 | 1019.02 | 1.01 | 0.31 |
|  | group*frequency | 2 | 1019.13 | 0.27 | 0.76 |
|  | stimulus*frequency | 2 | 1019.02 | 23.83 | **< 0.001** |
|  | group*stimulus*frequency | 2 | 1019.02 | 1.21 | 0.30 |
| Pain unpleasantness | group | 1 | 35.03 | 0.01 | 0.93 |
|  | stimulus | 1 | 1019.02 | 439.80 | **< 0.001** |
|  | frequency | 2 | 1019.12 | 40.58 | **< 0.001** |
|  | group*stimulus | 1 | 1019.02 | 8.27 | **< 0.01** |
|  | group*frequency | 2 | 1019.12 | 0.32 | 0.73 |
|  | stimulus*frequency | 2 | 1019.02 | 36.93 | **< 0.001** |
|  | group*stimulus*frequency | 2 | 1019.02 | 1.92 | 0.15 |
| Reflex responses | group | 1 | 30.19 | 3.01 | 0.09 |
|  | stimulus | 1 | 881.24 | 38.09 | **< 0.001** |
|  | frequency | 2 | 881.70 | 25.03 | **< 0.001** |
|  | stimulation intensity | 1 | 29.97 | 2.98 | 0.09 |
|  | group*stimulus | 1 | 881.24 | 0.14 | 0.70 |
|  | group*frequency | 2 | 881.67 | 0.42 | 0.66 |
|  | stimulus*frequency | 2 | 881.24 | 24.05 | **< 0.001** |
|  | group*stimulus*frequency | 2 | 881.24 | 0.95 | 0.39 |
| Statterwaite’s method was used to estimate degree of freedoms (df), *F* and *p* values, as implemented in the *R* package *lmerTest* ; ANOVA = analysis of variance | | | | | |

1. **Correlation between temporal summation of pain perception and reflex responses in participants with BPD and NCC**

| ***Table S7:*** *Correlation between temporal summation of pain perception and reflex responses in participants with borderline personality disorder and non-clinical controls* | | | |
| --- | --- | --- | --- |
|  |  | **Temporal summation pain intensity** | **Temporal summation pain unpleasantness** |
| **Temporal summation reflex response** | **NCC** | *r_s_* = 0.57, ***p* < 0.05** | *r_s_* = 0.50, ***p* < 0.05** |
|  | **BPD** | *r_s_* = 0.35, *p* = 0.20 | *r_s_* = -0.12, *p* = 0.67 |
| NCC = non-clinical controls; BPD = borderline personality disorder | | | |
